# From Product to Place—Spatializing governance in a commodified landscape

**DOI:** 10.1007/s00267-017-0883-7

**Published:** 2017-05-24

**Authors:** Cora van Oosten, Moira Moeliono, Freerk Wiersum

**Affiliations:** 1 Wageningen UR Centre for Development Innovation, P.O. Box 88, Wageningen, AB 6700 Netherlands; 2 CIFOR International Center of Forestry Research, P.O. Box 0113 BOCBD, Bogor, 16000 Indonesia; 3 Wageningen UR Forest and Nature Conservation Policy research group, P.O. box 47, Wageningen, AA 6700 The Netherlands

**Keywords:** Landscape, Governance, Oil palm, Multifunctional concessions, Policy integration, Indonesia

## Abstract

This article analyzes the potential for landscape governance in large-scale commodity landscapes in Indonesia. It conceptualizes landscape governance as the spatialization of governance, which entails the interplay between natural-spatial conditions of place, public-private actor constellations, and policy responses. The article presents the case of a commodified oil palm landscape in West Kalimantan, where a potentially new type of landscape governance is emerging out of the experimental activities of an ecologically responsible commercial enterprise. It describes the development of a multifunctional concession as a process of productive bricolage involving the creative combination of different land uses within a single productive space. It also describes how such a multifunctional concession does not fit into existing policies, which are sectorally defined and embedded in sticky institutional frames. The formation of new public–private institutional arrangements needed for the development of multifunctional concessions is a difficult process, as it requires an alignment of contrasting discourses and an integration of sectorally-defined policy frames. If successful, it might facilitate the transition from multifunctional concessions to multifunctional landscapes. Such a fundamental change in land use and production relations however requires intensive stakeholder engagement and policy dialog. Indonesia’s continuous decentralization process offers opportunities for this, as it increasingly provides institutional space at the landscape level, for public and private actors to explore common concerns, and craft public–private arrangements specific to the landscape.

## Introduction

In response to global agreements combating climate change, in particular the most recent sessions of the Conference of the Parties of the United Nations Framework Convention on Climate, landscape approaches are gaining popularity worldwide. As a means of reconciling forest conservation, agricultural production and livelihood options (Rahman et al. 2015), but also as a way to combine public and private interests, promote stakeholder collaboration within commodity chains, and highlight the importance of placing commodity chain performance within a place-based or landscape perspective (Ros-Tonen et al. 2015). Landscape governance has been defined as the process of multi-sector, multi-actor and multi-level interaction and decision making at the landscape level (Colfer 2011; van Oosten et al. 2014; Ros-Tonen et al. 2015; Kusters 2015). It is in this context that Sayer et al. (2013) developed a set of design principles to guide landscape-level decision-making processes in a democratic, transparent and informed way, taking into account the interests of the various stakeholders involved. One instrument often proposed to enhance landscape governance is the establishment of platforms for public-private^1^ dialogue. Such platforms offer a means to harmonize stakeholders’ views and interests, and embark upon a process of joint planning (Kozar et al. 2014; Kusters 2015). Creating a platform, however, is only one aspect of landscape governance, and will only be beneficial if it forms part of a larger process of developing new institutional mechanisms for stakeholders to meet, deliberate, align discourses, and embark upon a process of shared learning (Van Oosten 2013; Van Oosten et al. 2014).

The development of new institutional mechanisms not only relates to the process of governance, but also to the object to be governed—which is the landscape (Van Oosten et al. 2014, building upon Kooiman 2003, 2008). This substantive component of governance has a threefold importance. Firstly, landscapes are not static objects, but rather dynamic, due to the nature of the spatial processes they incorporate. Secondly, the characteristics of a landscape are perceived differently by the various stakeholders involved (Van Oosten 2013; Van Oosten et al. 2014). These perceptions are often based on their interests or “stakes” as well as formal sectoral considerations and policy frames defining the relations between the actors and the landscape. Finally, the specific landscape dynamics as perceived by actors influence the ways in which these actors interact and make decisions. This interplay between the social and biophysical dimensions of the landscape shapes both the landscape and the actors, and new forms of landscape governance may therefore require political and institutional reform.

To illustrate this interdependency between the substantive matter and the process of governance, we will present a case study from Indonesia—which is a country with one of the world’s highest deforestation rates, largely due to the rapidly expanding palm oil industry (Sirait 2009). Societal criticism has only recently forced the palm oil industry to admit it’s devastating impact on forests, and publicly pledge to decrease or halt deforestation (Pirard et al. 2015). In order to realize these pledges, several palm oil producing companies have become supporters of sustainability and zero-deforestation movements and started searching for alternative production models that are more sensitive to the ecological conditions within their sourcing areas.

### The aim of this article

This article aims to contribute to the understanding of the complexity of landscape governance as a combination of novel land use practices and institutional bricolage. This requires a *new institutionality*, which stimulates the creation of novel public-private governance arrangements at the landscape level (Van Oosten et al. 2014). A major question is how these public-private arrangements are shaped in practice, considering that the two actor categories have different relations to the landscape; relations which are discursively embedded and shaped through different institutional frames. To address this question, we focus on the commodified oil palm landscape of West Kalimantan in Indonesia. We present a case study on one company which is in the process of developing an innovative production model for its concession^2^. The case does not present a “best practice” but illustrates the emergence of a new trend of ecologically responsible companies, proposing a more creative use of their productive space through the design of multifunctional concessions. These multifunctional concessions however do not fit within existing policy frames, which are embedded in sectorally defined, inflexible or “sticky” institutions (Hajer 2003). Thus, it is an open question whether these private initiatives can lead to the required institutional change for more sustainable production and more inclusive spatial decision-making. In brief, this article addresses the following questions:

1. How did the West Kalimantan concession landscape emerge out of the interplay between its natural and its socially constructed conditions of place?
2. What changes in institutional arrangements occurred in the development of West Kalimantan’s concession landscapes and how are these discursively embedded?
3. What was the outcome of the novel multifunctional concession design ? 

## Analytical Framework: Landscape Governance Unraveled

### Landscape Governance as a Process of Spatialization

One of the first authors to systematically conceptualize landscape governance is Christoph Görg (2007). He characterizes landscape governance as the interconnections between socially constructed spaces and the natural conditions of places. He argues that today’s complex environmental problems are anchored in particular places yet have a global impact. This local-to-global relationship requires a system of governance, which links the spatial characteristics of place with higher scales of political decision-making. He highlights these ‘politics of scale’ by outlining how this involves a restructuring of the spatial organization of states. As the political and economic processes no longer overlap in spatial coverage, shifts between private and public regulatory areas and between the relationships of market processes and their political regulations are needed. As a result, new constellations of actors are emerging including non-state actors such as civil society movements and private companies, each having their own spatial reference regarding both the landscape and the political decision-making process. Görg argues that due to this separation of the locus of spatial decision-making and the source of commodities, newly emerging landscape governance arrangements may require policy responses beyond the current policy frames.

Görg identifies three key areas as determining the nature of landscape governance: (a) the natural conditions of place, (b) the public–private actor constellations, and (c) the policy responses. This conceptual model entails two crucial questions of how such spatialization of governance works in practice: How are these public–private actor constellations embedded in different discourses reflecting the difference between public and private use of space ? In addition, how do the different actors develop new institutional arrangements across multiple levels and scales ? 

### Changing Institutional Arrangements by Navigating Between Sectoral Discourses and Practices

The spatialization of landscape governance requires not only new actor constellations, but also new discourses and institutional reform. It is based on novel views on the desired nature and dynamics of the landscape and the segregation and integration of different land-use types, e.g. agriculture and nature, production and protection, mono- and multifunctionality of a landscape (Van Oosten et al. 2014). Integration of different land uses and their respective policies requires “navigating” between land-use sectors, and between local, regional, national and supranational scales of spatial decision-making. Such navigation implies that landscape governance is based on discourses as ‘interpretative schemes, ranging from formal policy concepts and texts to popular narratives and storylines giving meaning to a policy issue’ (Arts and Buizer 2009, Buizer et al. 2016, page 4). The interplay between different discourses on which the institutionalized practices of the various stakeholders are based is inherently part of the spatialization, or integration of institutional practices within place (ibid).

Landscape complexity also requires new governance arrangements that transcend existing institutional boundaries (Hajer 2003). New actor constellations need to negotiate new rules and behaviors regarding the space in which they operate. It is this “new spatiality” (ibid.) that demands actors be able to “jump scale” not only in terms of territoriality, but also in terms of sticky institutional structures. This requires a process of crafting new institutional arrangements out of “old” sectoral policy frames and “new” place-specific arrangements.

### Landscape Governance as a Process of Institutional and Productive Bricolage

Cleaver (2002, 2012) characterized the process of crafting and reconstructing institutions as “institutional bricolage”. The term institutional bricolage refers to the dynamic and ad hoc flexible nature of the governance process in the form of (re)constructing institutions, pieced together by individuals acting within the bounds of circumstantial constraints. The outcome of this process is often unforeseen, as much depends on the power relations between the different actors involved, and their respective agency as bricoleurs (Cleaver 2012; De Koning 2014; De Koning and Cleaver 2012; Funder and Marani 2015). Ros-Tonen (2012) introduced another form of bricolage which she calls “productive bricolage”, referring to the “flexible and dynamic crafting together of various livelihood options and its associated impacts on the landscape” (p. 17). In proposing this term, she refers to Madge (1994) who describes how various land-based activities are combined as a strategy of local communities to cope with external stresses. She also builds on Batterbury (2001) who describes productive bricolage as a dynamic process, not only to cope with stresses, but also to grasp opportunities, and creatively build economic diversity at the local level. Ros-Tonen (2012) identifies this process of economic diversification as not only involving “diversification by necessity”, as response to external forces, but also “diversification by choice”, emerging from the multi-scalar interactions between the various actors involved. In this way, we consider the concepts of institutional and productive bricolage as complementary in enabling the diversification of production models within the overall institutional landscape.

Although productive bricolage has predominantly been identified in relation to the land-use systems of local communities, it can also be applied to landscapes managed by local bureaucrats who negotiate their position between local communities and the central state by using both formal procedures and informal practical norms (Funder and Marani 2015; Kubo 2010). Such productive bricolage may also be applied by private companies which respond to changing market and policy conditions, e.g., in the form of increased demand for sustainably sourced commodities. This may challenge the justification of monotonous “commodity-scapes” having high yields yet low bio-cultural diversity, and encourage more innovative sourcing strategies for combining commercial production, environmental conservation and communities’ well-being through a clever integration of land use within a single space (Koh et al. 2009; Santika et al. 2015). Such innovative sourcing strategies may include a combination of productive and protective zones, including multifunctional agroforestry areas, and corridors between high-conservation-value forests (Koh at al. 2009). Experiments in Brazil show that through the development of more land- and labor-efficient production techniques and more inclusive management, diversified concessions are feasible (Brandao and Schoneveld 2015). Though financial returns may decline, this may be compensated through the avoided costs of environmental degradation and social unrest (ibid). In the long run, productive bricolage practiced by companies can be economically feasible, and socially and ecologically desirable as an alternative to the current unsustainable business practices.

## Study Area and Methodology

West Kalimantan is one of five^3^ provinces of the Indonesian part of Borneo. It covers approximately 14 million ha, and is inhabited by 4 million people, the majority living in rural areas (Sirait 2009). Rubber and palm oil are the most important export commodities, with over 0,35 million ha planted with oil palm (Potter 2008; Colchester et al. 2006). In addition, a large area has been cleared for palm oil production but not yet planted. West Kalimantan’s contribution to Indonesia’s palm oil production is estimated at 6% (USDA 2010), a figure which, despite the recently announced moratorium on new palm oil expansion, is likely to grow (Jong 2015, Diela 2016). This trend is of great concern in view of the environmental effects of deforestation, and has led to the development of novel ideas on mixed concession landscapes including both commodity plantations and conservation areas (Pirard et al. 2015).

We use an explanatory single case study approach, to assess the context specificity of landscape governance. Such a qualitative case study approach is useful to understand the relationship between the social phenomenon and the context in which the phenomenon occurs (Yin 2009). We have taken the commodified landscape of Ketapang, on Indonesia’s West Kalimantan, as an example. Here, we conducted a series of studies to assess the potential of multifunctional concession landscapes, beginning with an overview of the history and recent developments of the West Kalimantan commodified landscape. Second, a study was conducted on the current production models of West Kalimantan’s palm oil industry, and their potential for innovation. Our case study in the Ketapang district of one particular palm oil company experimenting with a multifunctional concession design was purposely selected as an example of a new practice approach in the making. A final study focused on the institutional framework surrounding palm oil production in Indonesia, and the analysis of institutions hampering multifunctional concession design. The various studies were complemented and triangulated by empirical data based on interviews with private and public stakeholders, as well as with community members and leaders inside and outside the concession area. In 2014, a first series of 25 interviews were conducted amongst local inhabitants, rubber and palm oil farmers, processing companies, middlemen, NGOs and government officials in West Kalimantan. Additional interviews were held with CIFOR (Center for International Forest Research) scientists in 2014, 2015, and 2016. All interviews were documented in interview reports and analyzed in a qualitative manner, to systematically assess stakeholder dynamics and institutional processes. The results were discussed and critically evaluated in the context of two international meetings in Indonesia organized by Wageningen UR and CIFOR (2015,2016)^4^, and a public seminar in Wageningen in 2016.

## Results: The Process of Developing a New Multifunctional Concession Landscape

The process of developing a new multifunctional concession landscape is presented in three sections, corresponding to the three research questions presented in Section 1

### The West Kalimantan Concession Landscape as the Product of Natural and Socially Constructed Conditions of Place

#### The historic formation of West Kalimantan’s landscapes

West Kalimantan’s landscapes have been historically shaped by their natural conditions, forming the basis of its production systems, which have in turn been subject to political trends, legal systems and markets. Traditionally, the population of West Kalimantan, known as the “Dayak”, named their landscapes after the rivers and dominant tree species. An example is Ketapang, the name of our study district, named after the Terminalia catappa tree, which is quite common in the area. Originally, the Dayak built their livelihoods on swidden agriculture, supplemented by hunting and gathering forest produce; while commercial agriculture and trade were mainly carried out by the Malay and Chinese population living along the coast. During the colonial period, West Kalimantan gradually entered the global market. At the beginning of the 20th century, the Dutch colonizers introduced the rubber tree, originally from South America, with the aim of setting up plantations feeding into the market of the then-industrializing world. Initially, the rubber tree was not much appreciated by the local population. However, rubber became an unintentional means for the Dayak to acquire land rights (Peluso 2009). Under colonial rule, the Dayak people could occupy and access “customary land” if they could prove that they were actually using it. Within the swidden agricultural system, it was hard to prove land use, unless the swidden was “tagged” with productive trees. This is how the Dayak people adopted the rubber tree, first as a means to “tag” their plots, and later as an easy way to gain a monetary income. Rubber became popular, and West Kalimantan became the heartland of the rubber industry, dominated by smallholder production. The complex trading system dominated by middlemen formed a complex social structure of interdependent relations (interviews with local people, confirming earlier findings of Peluso in 2009 and Sirait in 2009). The resulting rubber gardens or agroforests were and still are highly biodiverse, providing rural families with a diversified livelihood and collective identity, and contributing to a resilient socio-ecological system (Joshi et al. 2002). During field research, farmers remarked that although it is currently the oil palm that they prefer because of its price, they retain a deep-seated bond with the rubber tree: ‘Where palm oil provides us with our daily rice, the rubber forests provide us with our savings account’ (quote of a local respondent).

#### The commodification of West Kalimantan’s landscapes

The introduction of the timber, rubber and mining industry since colonial times changed perceptions of forests and the value of forestland (Barr et al. 2006). This process accelerated with the introduction of the first oil palm plantations which were established in West Kalimantan in the 1980s (Barr et al. 2006; Sirait 2009). Oil palm plantations were initially introduced as government enterprises using the “nucleus plasma” model, in which the enterprises formed the “nucleus”, while smallholdings constituted the surrounding “plasma” (Acciaioli 2016). However, during the structural adjustment period in the 1990s, the plantations were privatized and purchased, mainly by multinational corporations. Production, trade and processing became concentrated in horizontally- and vertically integrated conglomerates or business groups, often run as joint ventures with foreign investors (ibid.). Government policies facilitated large-scale oil palm expansion through cheap land concessions, state bank loans to companies, and state-organized transmigration programs to provide cheap labor. This expansion came at considerable cost to forests, agroforests and often in conflict with local communities and forest owners (Sheil et al. 2009). More recently, decentralization shifted the responsibility of issuing production permits to the local authorities. This change involved an implicit acknowledgement of community rights as the companies had to directly negotiate with communities to acquire user rights to the land. From 1980 to 2009, West Kalimantan saw a ten-fold increase in palm oil production (Sirait 2009), often expanding to forest and peat lands. Forest conversion went hand in hand with unconstrained forest exploitation, fires and road building, and massive drainage of peat lands caused high emissions of carbon dioxide. In our study district Ketapang, 70% of the land has been licensed to corporate plantation developers. The district government has issued 39 oil palm permits that fully or partially overlap with 400,000 ha of protected forestland (ibid.).

#### The growing disconnect between people and place

Some community leaders told us that they were fully aware of the ongoing land conversion; others told us they discovered their land had been allocated to oil palm companies without their knowledge. At the end of 2008, there were at least 20 major land conflicts in Ketapang district alone (Zakaria et al. 2009). Most of these conflicts are related to land registration, land conversion, and negotiation over contract conditions (Rietberg 2011). In many cases, the legal process has been correctly implemented, but the process itself reflects the transformation from a dynamic to a static system of tenure rights, creating more exclusive forms of rights over resources (Meinzen-Dick & Mwangi 2009; quoted by Rietberg 2011). There are also conflicts over financial returns and additional benefits as agreed in the contract (roads, public facilities) and discontent due to misaligned expectations at the outset of the process (Rietberg 2011).

### The Discursive Embeddedness of Changing Institutional Arrangements

Ideas regarding commodified landscapes started to change around 2010, triggered by unprecedented annual fire and haze disasters. The *New York Declaration on Forests* (2014) represents a call to action by a group of leading international corporations. One of its commitments is to *at least halve the rate of loss of natural forests globally by 2020 and strive to end natural forest loss by 2030, and support and help meet the private-sector goal of eliminating deforestation from the production of agricultural commodities by no later than 2020 s* (New York Declaration on Forests 2014). Although the declaration is a non-legally binding document, it does commit its signatories to drastically change the way in which they do business. As a result, the “zero deforestation movement” was born, representing a private sector-led initiative to eradicate deforestation from their operations and commodity chains (Fishman 2014).

The Indonesian Chamber of Commerce and Industry representing Indonesian commodity companies embraced this movement, and many companies pledged considerable contributions. These pledges positively influenced the ongoing negotiations between the palm oil industry and civil society organizations at both the Round Table on Sustainable Palm Oil (RSPO); and the Indonesian Round Table on Sustainable Palm Oil (ISPO), which are less far-reaching than the RSPO guidelines, but mandatory for all oil palm companies operating in Indonesia (Pirard et al. 2015).

Despite these achievements, there have been concerns about the general neglect of the RSPO/ISPO standards to what is happening “upstream in the commodity chain”, particularly in relation to deforestation and the poorly defined land tenure systems (Brassett et al. 2011; Van Bodegom 2013). In response to these concerns, in 2013 a group of leading producing companies initiated the Indonesian Palm Oil Innovation Group (POIG) published a *No Deforestation, No Peat, No Exploitation Pledge* (Fishman 2014; Pirard et al. 2015), which embraces a landscape approach, in the sense that it recognizes the importance of forest conservation within concession areas. This was directly following a proposal of Indonesia’s largest palm oil-buying companies, obliging their suppliers to assure that palm oil operations have no deforestation footprint. Although there is no clarity as to what “zero deforestation” means in real and measurable terms, several producing companies have started assessing “high carbon stocks” and “high conservation value forests” within their sourcing areas, and identifying potential set-asides for compensation. They also started community consultations in various high-conflict zones, and the development of sustainable peatland management plans. All these measures potentially affect companies’ modes of operation, and make them more sensitive to the “socio-ecological characteristics of place”. The critical point lies within the operationalization of the commitments made, but there is overall optimism regarding the seriousness with which measures are being taken, implemented, and monitored (ibid.).

#### A novel landscape proposition: multifunctional concession design

The way in which companies aim to operationalize a landscape approach varies considerably. Incentivised by consumers demanding more sustainable products and local communities demanding more inclusive business models, some private companies claim to have adopted a landscape approach because they have set aside land outside of the concession areas to compensate for forest loss within concessions. Others claim to have adopted a landscape approach because they have swapped high conservation value forests within their concessions for degraded forestland outside of their concession area to be taken into production (Leone 2015; Pirard et al. 2015). In both cases, the measures are focused on enhancing multifunctionality of the landscape outside of the companies’ own productive space. More innovative are the companies, which decided to drastically change their concession design. With this, they try to comply with the governmental regulations on non-burning and conservation of riparian zones, but also to recognize the presence of high conservation value forests and multifunctional rubber gardens within their lease area. Adapting their production plan to this spatial reality creates more diverse and multifunctional production areas, but has implications for productivity and profitability. Some timber and paper enterprises experimented with such multifunctional concessions combining production and protection zones, benefiting commodity production, biodiversity and rural communities.^5^ Several NGOs supported the initiative to operationalize the Zero Deforestation pledges through such multifunctional concession design. Other NGOs however are more critical, saying that new production models, although well intended, do not fundamentally change production relations, especially with regard to land tenure.

The implementation of multifunctional palm oil concessions is not easy, as it requires working at the interface between agricultural and forestry laws. This can be illustrated by the experience of a young medium-sized Indonesian palm oil company, listed on the Singapore Exchange, in this article referred to as The Company.^6^ The majority of its palm oil is produced on The Company’s own plantations and associated plasma areas, while approximately a quarter (24.7%) is derived from nearby independent smallholders and outgrowers. In reaction to a series of formal complaints from NGOs on illegal clearing, The Company has built upon the RSPO principles in formulating a new sustainability policy. This policy states that *we need to develop a strong integrated landscape approach to ensure that natural habitats are protected whilst not depriving local populations from meeting their development needs. Our holistic approach provides for the protection, restoration, compensation and/or co-management of forests and other areas identified as having high conservation value. We are trialling participatory landscape approaches within and around the boundaries of our plantation lease areas to promote conservation and sustainable use of forest, peat, agroforestry with oil palm plantations to promote diverse landscapes that contribute to long term food and income security. Our exploration of options places the village level at the centre of decision-making* (The Company’s Sustainability Report 2014).

Since the revision of its Sustainability Policy, free, prior and informed consent has become part of The Company’s operational procedure, to openly dialog with communities and individuals whose customary land claims are impacted by the Company’s concessions. The Company reserved one entire concession of 16,900 ha for the real-life development of a multifunctional concession design to explore, develop and test a more sustainable and inclusive production model. The new concession design shows a much larger variety of land uses than its original block pattern design. Whereas in the original design, 12,500 ha (74%) of the total concession area was planned to be planted with oil palm, in the adapted design there is only space for 6581 ha (39%) of oil palm plantations. The other 61% of the concession area is allotted to conservation forest, rubber agro-forest, protected riparian zones, and cultural-spiritual sites, which offer local communities the option to maintain the multifunctional character of their production system, and keep their rubber agro-forests intact. The fundamental difference between the old and the new design, is that the new design departs from the common block division but uses much more natural features and incorporates a much larger variety of land uses. As well it is developed in consultation with the communities inside or surrounding the concession area. All ongoing land acquisitions were put on hold while a multi-stakeholder negotiation process on access rights was initiated. This, to the content of community members who did not want to sell; yet to the discontent of others who had wanted to sell and move to the city. While the negotiation process is ongoing, the alternative design is being assessed for its technical and financial viability, social acceptability and ecological impact. Initial studies confirm that the alternative design reduces The Company’s income from oil palm considerably. However, the costs of resource degradation and social unrest are also expected to decline. Although it might be naive to assume that the opportunity costs will compensate for the decline in oil palm income, the novel concession design opens new vistas to explore alternative incomes to be derived from the concession (Molenaar et al. 2011; Joshi et al. 2002; Wibawa et al. 2006; Saavedra and Guijt 2015). It stimulates the development of multiple-product business models, including NTFPs (such as rubber) and carbon sequestration (ibid). It is predicted to also stimulate the formation of new business alliances with smallholders and outgrowers, as well as with other industries such as the rubber industry. This would allow for a combination or integration of production systems on a larger scale. Such collaborative landscape design has the potential to create space for conservation areas between production units to serve as ecological corridors, thus combining production and protection at the macro-landscape level.

The operations of The Company may not be representative of all palm oil companies in the area. But it does show the example of a pioneer company, which seems genuinely motivated to learn from past mistakes, and search for alternative pathways to change mainstream production models into more sustainable and inclusive ones.

### The Outcomes of the Multifunctional Concession Design: An Institutional Mismatch

In principle, multifunctional concessions fit into the historically developed landscapes of multifunctional production systems based on productive bricolage by local households. Its development would increase the social acceptability and environmental sustainability of the palm oil industry. Multifunctional concessions would tailor well with the increasing number of smallholders engaged in palm oil production, either through the nucleus plasma system, or as independent smallholders. Nonetheless, their feasibility is still uncertain, as the financial viability of multifunctional concession design still has to be proven. Moreover, institutional feasibility seems very complex.

#### Fitting multifunctional concession landscapes in existing legal frameworks

Although multifunctional concessions fit into the traditionally shaped landscape, they do not fit into the existing legal frameworks regarding agrarian and forestland use. The higher the multifunctionality of the concession, the more complex becomes its legal embeddedness, as the various land-use components fall under different legal domains. This was illustrated by respondents from different ministries who gave substantially different answers to the question as to which law is responsible for regulating palm oil concessions. Palm oil production in Indonesia is formally under the purview of the Directorate General of Plantations, under the Ministry of Agriculture. However, as oil palm concessions usually harbor various types of land cover, the concession holders must comply with agrarian, forest, environmental and spatial planning laws, which have different perspectives regarding land use (Leone 2015; Suryadi 2015). The agrarian law provides the basic rules for obtaining a location permit on land designated for agricultural use. However, if the permit issued contains forestland, the status of the land has to be changed under the forestry law. The environmental law provides regulation on environmental impact assessment, management and control. The spatial planning law regulates land-use systems at national, provincial and district level. The different regulations, implemented through different ministries, are often in conflict. For instance, the Ministry of Agrarian and Spatial Planning recently issued a circular (10/SE/VII/2015) instructing local governments to stop issuing concession permits for areas of high conservation value forests outside the designated state forest and to prevent clearing of these forests. This letter is in opposition to an earlier regulation requiring complete cultivation of the area covered by the concession permit within 6 years (Leone 2015). Whereas this regulation was originally meant to avoid land speculation, it is currently hampering the development of more multifunctional production models.

#### The influence of multi-level governmental institutions

Considering the complex and sometimes conflicting legal requirements, the development of multifunctional concessions not only requires productive bricolage, but also institutional bricolage. Such institutional bricolage not only relates to the crafting of new interfaces between the requirements of multiple legal frameworks, but also to the development of new roles and responsibilities of government institutions operating at different levels. Within the context of decentralization, local governments are increasingly allowed to “bricole” within the spirit of the different laws, provided they avoid negative socio-ecological impacts (Barr et al. 2006). Such ‘institutional bricolage’ is legal, as local authorities are legally mandated to adapt spatial and environmental law if it helps to reduce environmental degradation or social unrest (Leone 2015). The decentralization law of 2014 (Law 23), although re-centralizing part of the authority over forests at the provincial level, did not change much in the agriculture sector (Simarmata and Firdaus 2016; Steni 2016). Thus, oil palm plantations will still be governed by the district if contained within one district; or by the province if it straddles more than one district. That said, the new law on villages (Law 6, 2014) gives full authority to village governments to manage their ‘assets’ which include village land. According to respondents, Ketapang’s District Government is known as being progressive, using its mandate to actively tailor the law to local circumstances, facilitate public–private- dialog, and mediate in company-community conflict. However, the District Government also needs revenues to respond to the demands of their constituencies, which is most easily obtained through the levies raised by concessions. This dual interest makes it hard for The Company to negotiate with the District Government and have its production plans approved.

Efforts to craft new institutional arrangements at the interface of land use regulation and the raising of government revenue become more problematic at the level of the Central Government. The government has stated its opposition to the Zero Deforestation movement and announced a presidential directive that would serve as the legal basis for a 5-year moratorium on new palm oil concessions (Diela 2016). As a representative of the Ministry of Economic Affairs has publicly argued, the most effective driver of economic growth in Indonesia is the palm oil sector; operationalizing the Zero Deforestation pledges would jeopardize the country’s economic growth. He also argued that raising operational standards for palm oil production will put restrictions on the growing group of smallholder producers and cause problems for smaller palm oil firms in their commodity chain (Taylor 2015). Officials even fear the emergence of cartel practices, encouraged by deliberately setting standards too high for smallholders to comply with (Shenq 2016). Despite the contribution of smallholders to deforestation, the government will continue to protect them in order to avoid them being driven out of business through standards set by foreign-owned buying companies (Jong, in Jakarta Post, August 29th 2015). A second argument against the Zero Deforestation pledges is that the government considers them to be too much of a proactive private sector engagement in policy reform. According to the Ministry of Environment and Forestry, the scope of the pledges interferes with the authority of the Government, therefore breaching the State Constitution. According to a representative of the Ministry, the Government risks losing sovereignty when its authority is taken over by the private sector (Jong, in: Jakarta Post, 29 August 2015). This stand reflects the government’s discomfort with the idea that non-state actors (Pirard et al. 2015) can govern land use within private concessions. As a result, some of the largest palm oil companies decided to backtrack on their Zero-Deforestation pledges. They realized that if the government is really taking on a more active role in shaping the palm oil industry, working against the government will be counterproductive; cooperating with the government would be a more constructive course (Shenq 2016).

## Discussion: Spatialization of Governance as a Process of Productive and Institutional Bricolage

Our case study illustrates the importance of addressing landscape governance as a place and context specific process. It shows how the process of governance depends on its substance, that is, the historically grown spatial conditions of place.

Referring to the first research question, *how did the West Kalimantan concession landscape emerge out of the interplay between its natural and its socially- constructed conditions of place*, our article illustrates Görg’s theory that landscapes have been shaped through the interplay between its natural and socially-constructed conditions of place. However, the influence of the natural and the socially- constructed conditions has never been equal, and the balance between the two has shifted over time. The pre-colonial landscape was a product of rich ecosystems and the Dayak production system of swidden agriculture that shaped a bioculturally diverse landscape. During the colonial period, the introduction of alien commercial crops changed the landscape, opening the way to global markets and changing land tenure arrangements. People-place relations were further changed due to the introduction of the oil palm, which led to land alienation and high numbers of land-related conflicts. Whereas natural conditions shaped the original indigenous production systems, it was the global political economy that shaped the transition to more commercially oriented production systems. The resulting monotonous commodity-scapes are far from the original bioculturally diverse landscapes.

Referring to the second research question, *what changes in institutional arrangements occurred in the development of West Kalimantan’s concession landscapes and how are these discursively embedded*, our data illustrate how landscapes are subject to changing relations between the public and the private sector. In precolonial times, the Dayak population shaped the landscape through their customs, traditions and livelihoods. During the colonial period, the government appropriated resources and delegated concession rights to private concession holders. After the colonial period, the Indonesian government maintained the concession model whereby corporations were given the right to exploit resources and provide revenues to the state. This concession model is based on the arguments that palm oil production requires large upfront investment and strong vertical integration because of the perishable nature of the product (Byerlee 2014; Deininger and Byerlee 2011). In fact, the concession model was maintained for its economic importance, and the existence of the Dayak and their claims on resources were systematically ignored. The tenure system transformed from a flexible system depending on a family’s needs, to a static system, depending on an individual’s or enterprise’s formal tenure rights (Rietberg 2011).

It is during the past few years that the discourse of the private sector has changed from solely efficient commodity production to environmentally and socially responsible production. This is reflected in the growing Zero Deforestation movement. Some authors argue that the Zero Deforestation movement has been a response to the absence of government regulation (Pirard et al. 2015). Others claim that it is the decentralization process itself that resulted in the incorporation of new players; and that in a context of weak states, corporations gained control and reframed their interests as responsible yet only superficially changed modes of production (Lemos and Agrawal 2006). The Zero Deforestation movement and its operationalization through multifunctional concession design indeed reflects an ecological modernist discourse striving for win-win solutions, satisfying both the market and the environment (Buizer and Kurtz 2016; Dryzek 2013). However, the government’s counter-discourse opposes more private sector involvement in spatial decision- making and argues for increased smallholder production instead. According to Indonesian politicians, the Zero Deforestation movement interferes with the authority of the government and its monopoly on spatial planning; hence, the government’s fear of loss of sovereignty, as it sees its authority being taken over by the private sector. This is why some palm oil companies withdrew their Zero Deforestation pledges, as they realized that a more pro-active government attitude towards balancing land-use regulations would be in their favor (Pirard et al. 2015; Shenq 2016). This could potentially be the beginning of more mutual understanding and even more collaborative relations between state and non-state actors, based on the belief that both have a legitimate role to play as co-governors (Pirard et al. 2015).

Referring to the third research question, *what was the outcome of the novel multifunctional concession design*, we have seen that multifunctional concession design fits within the traditional multifunctional landscapes of West Kalimantan. However, it does not fit into the modern institutional framework surrounding palm oil production, which, despite some recent adjustments, is still sectorally defined, and has not allowed any form of multifunctional land use within the boundaries of a concession. If The Company implements its multifunctional concession design, it risks losing its concession, as it does not comply with any of the laws regarding oil palm plantations. In order to make it fit, a process of institutional bricolage as suggested by Cleaver (2002, 2012) would be required, to challenge existing policy frames and political power relations. Considering Hajer’s thinking on institutional stickiness (2003) this is however not very likely to happen. Nevertheless, under the political decentralization process, discourses are changing, and districts are accorded a certain freedom to adapt rules and regulations to specific local circumstances. There is room to institutionally maneuver at the local level (Funder and Marani 2015), especially since the new decentralization law has given provinces more power of oversight (Simarmata and Firdaus 2016; Steni 2016). It does however require courage on the part of District officials to make use of this institutional freedom and divert from centrally defined policy pathways

From these observations it can be concluded that our case study reflects the notion of landscape governance as combining productive and institutional bricolage. It also shows that productive bricolage is much easier than institutional bricolage. Productive bricolage is not a strange concept in the context of West Kalimantan landscapes, as the Dayak population applied productive bricolage in shaping its indigenous production systems, and to acquire land rights. Also multifunctional concessions can be considered as productive bricolage (Ros-Tonen 2012), as they lead to new and more creative landscape configurations, which fit much better into the natural-spatial conditions of place. Following Ros-Tonen (2012), multifunctional concessions can be considered as productive bricolage by choice, as it reflects a voluntary attempt to diversify production, reinterpret the concession model, and enhance collaboration with smallholders. However, it can also be considered bricolage by necessity, as power relations have changed, and consumer demand, environmental damage and social unrest force companies to reconsider their production models (Pirard et al. 2015). In both cases, productive bricolage can only be successful if it goes hand-in-hand with institutional bricolage. This is not unlikely, as local governments are constantly adapting the centrally-defined policies to their landscape-specific circumstances (Funder and Marani 2015). Nevertheless, our case illustrates that the institutional bricolage required for legalizing novel production models is not an easy process. Not only because of the sticky sectoral policy frameworks, but also because the discourses of the private and the public sector are in such sharp contrast, that a constructive public-private dialog seems hard to achieve. Where Görg (2007) sees the need for a change in the relationship between market processes and their political regulations, Ros-Tonen (2012) points to the lack of institutional interactions between administrative scales. The “new institutional spatiality” referred to by Hajer (2003), demands actors be able to “jump scale” not only in respect of territoriality, but also in respect of sticky policy frameworks. This requires more than just a process of crafting new institutional arrangements out of “old” sectoral policy frames, but also the balancing of rights, responsibilities and power positions of different actor categories sharing a single space.

### Scaling Up: From Multifunctional Concessions to Multifunctional Landscapes

Still, a classical concession implies a single-owner production model and unequal production relations between concession holders and local communities. Consequently, it is debatable whether the planning and management of multifunctional concessions can be conceived of as multi-actor landscape governance. However, the Company’s experimental concession design in principle provides space for a combination of both concession and smallholder production, allowing for multiple tenure arrangements and co-management of the area. Multifunctional concessions could therefore be considered a precursor to moving away from the classical mono-functional concession landscape towards a multifunctional landscape consisting of a diversified land use, ownership structure, and power relations adapted to a new local reality. This is in line with a global trend of moving away from large-scale concessions to collaborative smallholder production systems (Byerlee 2014). The management of The Company is not opposed to this trend, as it is well aware of its wider political ecology. It recognizes that currently 40% of the total palm oil production is estimated to come from “independent” smallholders (Budidarsono et al. 2015). This percentage is expected to grow given the strong government support for smallholder cooperatives. Thus, The Company is considering the option of concentrating on supporting production from smallholders rather than managing large concession areas with different types of land-use systems. This does not necessarily imply a weakening of The Company’s power position; it rather allows The Company to concentrate on its core business of producing and processing palm oil, which does not require land ownership per se. This could strengthen The Company’s collaboration with other commodity companies in for example rubber. Collaborating with other companies as well as smallholders would improve social relations and enhance sustainability. Such spatial transformation could simplify the presently complex governance arrangements on commercial and smallholder land-use systems and bridge the private and public sector discourses. This would not weaken but rather strengthen the role of the State, especially the District Government, in its role of facilitating landscape-level dialog, enabling the emergence of public–private partnerships specific to the landscape, and overseeing the level of inclusiveness of such new public-private partnerships.

## Conclusions

Since the emergence of international agreements to combat climate change, landscape approaches and landscape governance have received growing attention. Our case study illustrates that landscape approaches are gradually embraced by commodity companies and their proposition of combining productive plantations, smallholder production systems and conservation forests within a single space. Our study illustrates how these initiatives involve both a process of spatialization of production models, as well as changing relations between the private and the public sector. Due to its experimental nature, this process is characterized by both productive and institutional bricolage. However, the process of institutional reconfiguration is fraught with difficulties as a result of the persistence of competing discourses. Whereas private actors explore the opportunities of operating from a multi-sectoral landscape approach, public actors adhere to a sectoral orientation with a clear legal differentiation between agrarian production and forest conservation. Moreover, differences in opinion still exist in respect to the role of governments and private enterprises in land-use planning. Consequently, whereas multifunctional concession design can be regarded as successful productive bricolage, it is the institutional bricolage, or the creation of a “new institutional spatiality” which appears much harder to achieve. If landscape governance entails the creation of a new spatial reality embedded in spatially-integrated policies, it will take time.

This does not mean that multifunctional concession design is impossible. Currently, The Company and the District Government are negotiating the operationalization of one multifunctional concession on a pilot basis. Having a pilot status, The Company would be exempted from the general rules, to further test its viability. This provides space for combining productive and institutional bricolage, creating a new spatial reality,which reflects more diverse landscapes under multiple tenure arrangements Such new spatiality may fit better into existing policy frameworks, and offer space for smallholder production, and a mosaic of production models and tenure arrangements to coexist. This could be a precursor to more inclusive smallholder-dominated landscapes in future. However, we realize that our case study represents not more than a single case, the outcomes of which, especially in view of the institutional mismatch, may not lead to structural change.

In conclusion, we have seen that global environmental concerns have triggered the private sector to design innovative production models that better serve social and environmental interests. This can be interpreted as the ‘spatialization of production’ through productive bricolage by necessity and choice. This trend however has not yet led to a new institutionality, in which private and public actors jointly craft the institutional arrangements to give multifunctional concessions their license to operate. There is room for change at the landscape level, where a holistic approach would allow stakeholders sharing the same space to more easily come together to explore common concerns and align discourses. Indonesia’s decentralization policy does offer the institutional space for such exploration. But the actual use of this space depends on the ability of both the public and private sector to better align with the specific natural-spatial conditions of the landscape, and embark upon a process of public–private collaboration. This implies the capacity of all parties to understand each other’s interests, respect each other’s legitimate role as co-governors, and jointly create the appropriate mechanisms for landscape-level dialog to take place.

## Footnotes

1 With public we refer to governmental actors; with private we refer to non-governmental actors, such as private companies, citizens, and non-governmental organizations.

2 A concession is a contractual right to carry on a certain kind of business or activity, in this case palm oil, on government-owned land. Palm oil concessions in Indonesia are usually issued for a period of 30 years, which equals the life span of an oil palm.

3 In 2012 the province of North Kalimantan split from East Kalimantan

4 The international course on Landscape Governance is an annual course, which is jointly organized by Wageningen UR Centre for Development Innovation and CIFOR Center for International Forestry Research. Information can be found at https://www.wageningenur.nl/en/Expertise-Services/Research-Institutes/centre-for-development-innovation/short-courses.htm

5 See for example the experiences gained by the ‘New Generation Plantations’, see http://newgenerationplantations.org/

6 The Company is a member of the RSPO, has app. 200,000 ha under oil palm production mainly in Kalimantan and Sumatra, and owns eight processing mills, three of which are RSPO/ISPO certified. The Company has a “zero burning” and a “zero waste” policy, and has a relatively large percentage of its land under the nucleus plasma model.
